# Transfusion-Related Acute Lung Injury Type I Immediately after Open Surgical Repair for Abdominal Aortic Aneurysm

**DOI:** 10.3400/avd.cr.21-00143

**Published:** 2022-06-25

**Authors:** Kota Shimizu, Michihisa Umetsu, Hitoshi Goto, Takuya Fujimine, Daijirou Akamatsu, Takashi Kamei

**Affiliations:** 1 Division of Vascular Surgery, Department of Surgery, Tohoku University Hospital, Sendai, Miyagi, Japan; 2 Department of Vascular Surgery, South Miyagi Medical Center, Shibata-gun, Miyagi, Japan; 3 Division of Anesthesia, Kesennuma City Hospital, Kesennuma, Miyagi, Japan; 4 Department of Surgery, Tohoku University Hospital, Sendai, Miyagi, Japan

**Keywords:** transfusion-related acute lung injury, abdominal aortic aneurysm

## Abstract

A 68-year-old man underwent open surgical repair for an abdominal aortic aneurysm. The intraoperative period had no adverse events until postoperative extubation. His SpO_2_ rapidly dropped, and severe acute lung edema developed. He had cardiopulmonary arrest. Cardiogenic pulmonary edema and other diseases were ruled out. He was finally diagnosed with transfusion-related acute lung injury (TRALI) type I. Intensive care with ventilator management and continuous adrenaline administration was provided. His condition gradually improved, and he was discharged without any sequelae. Surgical cases requiring blood transfusion should be carefully monitored, and prompt action should be taken when TRALI occurs.

## Introduction

Transfusion-related acute lung injury (TRALI), defined as noncardiogenic pulmonary edema that occurs within 6 h of transfusion, is one of the most serious transfusion complications.^1)^ TRALI is relatively rare, accounting for approximately 0.4% of all transfusion-related adverse reactions.^2)^ However, the mortality rate is 5%–10%, and TRALI requires immediate treatment.^1,3)^ Here, we report a case of TRALI type I immediately after aortic replacement for abdominal aortic aneurysm (AAA). TRALI was successfully diagnosed and treated with mechanical ventilator management and continuous catecholamine administration.

## Case Report

**Figure 1:**
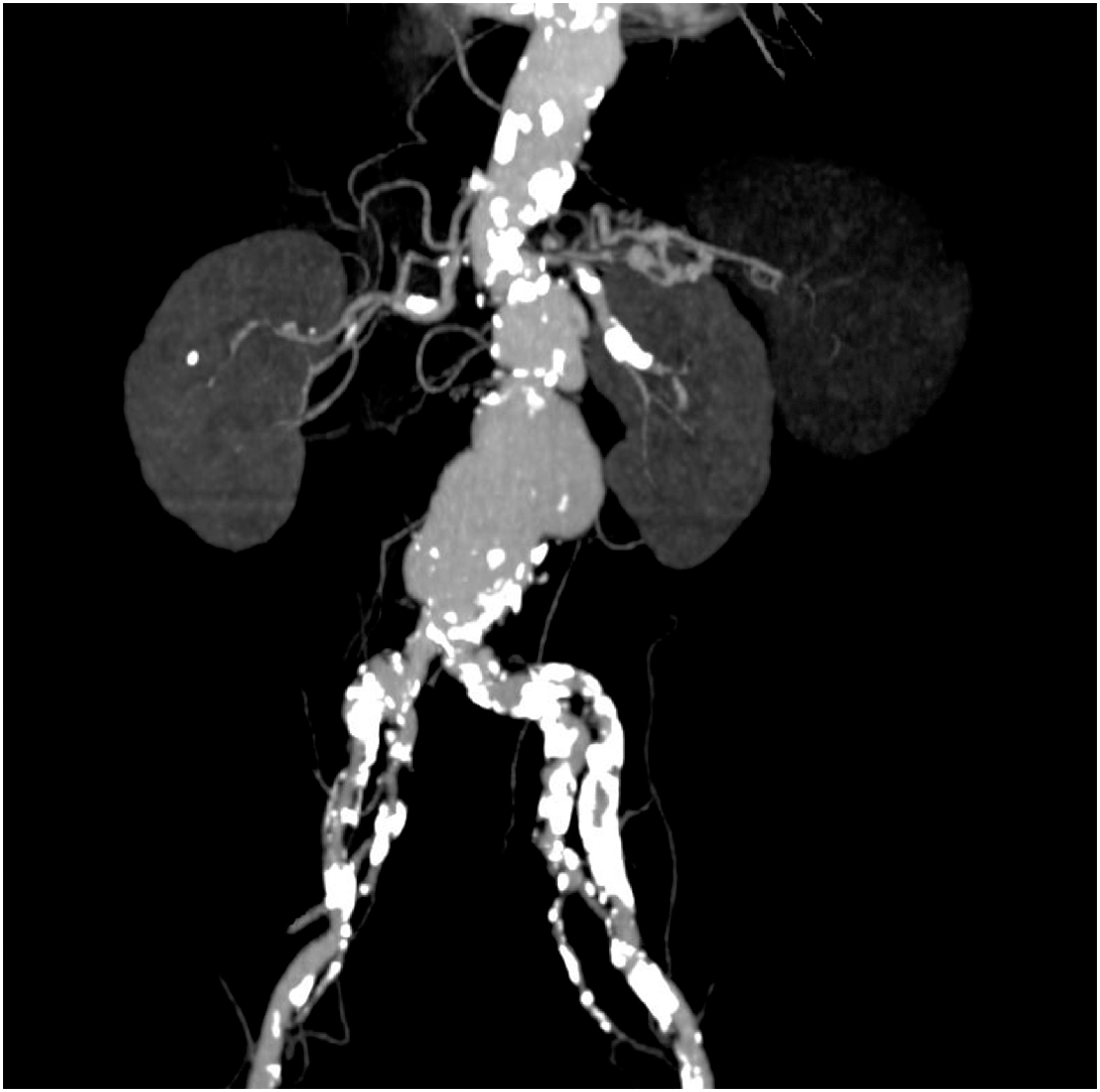
Fig. 1 Contrast-enhanced computed tomography of the aneurysm.

A 68-year-old man was incidentally diagnosed with a 68-mm AAA via computed tomography (CT; **Fig. 1**). The patient had a history of hypertension as well as a coronary artery bypass grafting (CABG) using the right gastroepiploic artery for angina pectoris 20 years prior. He was diagnosed with asthma because the fractional exhaled nitric oxide measurement performed to investigate the cause of his cough demonstrated a high level at 67 ppm, but he never had an attack. The patient was a current smoker and had no allergies. Preoperative chest radiographs revealed a mass in the right lung, which were previously observed but showed no changes in size or density. A preoperative blood examination revealed thrombocytopenia. The platelet count was 85,000/µL. The American Society of Anesthesiologists Physical Status (ASA-PS) was Class III due to angina pectoris. The blood group was type A, Rh-positive, and the screening test for irregular antibodies was negative.

Shaggy aorta and short proximal neck with severe calcification.

A contrast-enhanced CT scan revealed a shaggy aorta and a short-neck infrarenal AAA. Therefore, endovascular aortic repair was unsuitable, and an open surgical repair was planned. The preoperative examination was unremarkable, and long-term durability was expected.

Aortobilateral common iliac artery bypass was performed under general anesthesia using an 18×11-mm prosthetic graft (J-Graft, Japan Lifeline Inc., Tokyo, Japan). The operation time was 328 min, and the aortic clamping time was 100 min. Oozing was observed from the anastomotic site and retroperitoneal tissue due to thrombocytopenia and severe arterial calcification. Intraoperative bleeding was increased to 3,020 mL, which was stopped by intravenous protamine and compression. The total intraoperative infusion volume was 6,600 mL, including 1,400 mL of concentrated red cells and 720 mL of fresh frozen plasma. Because a cell saver was not available, allogeneic blood transfusions were performed. The PaO_2_/FiO_2_ ratio was consistently above 500 mmHg, and the intraoperative course had no adverse events.

**Figure 2:**
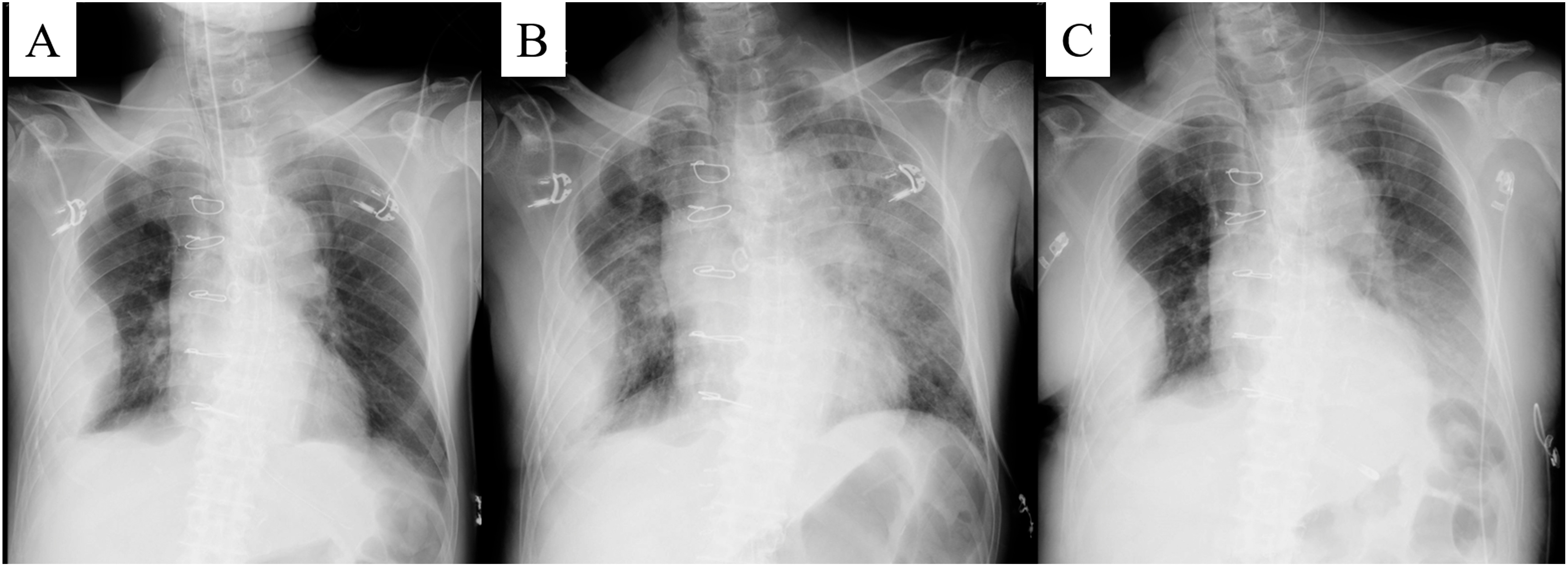
Fig. 2 Postoperative chest X-rays showing longitudinal changes.

The chest X-ray performed immediately after surgery revealed no significant findings, with the exception of the aforementioned mass (**Fig. 2A**). Tracheal extubation was performed after confirming that the patient was awake and the hemodynamic and respiratory status was within normal limits. The patient’s SpO_2_ dropped while preparing to leave the operating room, and his level of consciousness decreased. Fentanyl depression of respiration was considered, and a naloxone infusion as well as oxygen was provided. A nasal airway was inserted. Bag valve mask ventilation was stiff and resistant; thus, we suspected elevated airway pressure. The patient was re-intubated, and pure oxygen was supplied. The PaO_2_/FiO_2_ ratio dropped to 117 mmHg. Chest auscultation revealed pronounced bilateral wheezing, and it was considered that a major asthma attack could occur.

(**A**) Immediately after surgery: no congestive findings. (**B**) After worsening respiratory status and before intubation: marked pulmonary edema. (**C**) 9 days after surgery: pleural effusion remained but improved to the same level as before the surgery.

A chest X-ray revealed marked pulmonary edema (**Fig. 2B**). The patient gradually went into severe bradycardia and shock, and cardiopulmonary resuscitation (CPR) was initiated. The PaO_2_/FiO_2_ ratio decreased to 55 mmHg after 235 min from transfusion initiation and 115 min after its completion. The patient was easily resuscitated after the adrenaline administration. However, his heart rate and blood pressure decreased over time, and continuous adrenaline infusion at 0.1 µg/kg/min was initiated. The echocardiogram showed that cardiac contractility was maintained during bradycardia and after CPR. Thus, we agreed with the cardiologists that the patient did not have acute myocardial infarction or Takotsubo cardiomyopathy. The cardiologists also ruled out heart failure due to fluid overload because echocardiography revealed no evidence of right ventricular strain and pulmonary thromboembolism (PTE). We strongly suspected TRALI at this point, and 500 mg of methylprednisolone was administered. Respiratory and circulatory dynamics gradually improved, and the patient was transferred to his room under ventilator management. The N-terminal pro-brain natriuretic peptide (NT-proBNP) level was 741 pg/mL in the pre-transfusion sample and 564 pg/mL in the post-transfusion sample.

By the next morning, the PaO_2_/FiO_2_ ratio recovered to 300 mmHg. Catecholamines were subsequently decreased and terminated on day 7. The patient was extubated on day 9. Chest X-ray revealed improvement in pulmonary edema (**Fig. 2C**). The patient was discharged on the 31st day after surgery and was able to resume regular activities.

We reported the occurrence and the post-transfusion reactions to the Japanese Red Cross Society Blood Center. The patient was diagnosed with TRALI type I by several specialists. The NT-proBNP level before and after transfusion was the most significant finding indicating that the patient did not have right ventricular overload. The diagnosis was based on a combination of the absence of heart failure and other drug allergies that could have caused acute lung injury (ALI). All blood products used were negative for anti-human leukocyte antigen (HLA) antibodies. Both the transfused blood and patient samples were negative for Class I and II anti-HLA antibodies. No anti-granulocyte antibodies were detected.

## Discussion

We diagnosed and treated a case of TRALI type I immediately after open surgical repair of an AAA. Although cardiopulmonary arrest occurred, the patient was successfully rescued by continuous catecholamine administration and ventilator management. TRALI is defined as non-cardiogenic pulmonary edema that develops within 6 h of blood transfusion and is the leading cause of transfusion-related deaths according to the US Food and Drug Administration.^3)^ The Japanese Red Cross Blood Center reported that among cases of TRALI that occurred from 2012 to 2016, symptoms began within 2 h of the initiation of transfusion in 69.2%. The mortality rate for TRALI is 5%–10%.^1,3,4)^ The diagnostic criteria for TRALI were announced at a consensus conference and used worldwide.^1)^ The criteria were revised in 2019; the traditional TRALI was redefined as TRALI type I (without any risk factor for acute respiratory distress syndrome [ARDS]), and possible TRALI was redefined as TRALI type II (with an ARDS risk factor or with mild existing ARDS).^5)^

The incidence of TRALI was approximately 0.08% in patients who received blood transfusions, and TRALI accounted for 0.4% of transfusion-related adverse reactions.^2,6)^ Evidence reported TRALI occurring in the perioperative period,^7)^ and 38% of cases occurred in the perioperative period.^8)^ The diagnosis of TRALI requires the exclusion of other diseases that can cause ALI. In our country, TRALI is diagnosed at individual institutions, and the Blood Center conducts a TRALI survey by several experts to determine the presence of TRALI. Only 3–7 confirmed diagnoses of TRALI type I per year occurred from 2015 to 2019.

Perioperative diagnosis is difficult, and only a few reports mentioned TRALI occurring in the perioperative period of AAA repair.^9)^ We found no studies describing the differential at the onset of postoperative AAA as in this case; thus, this case represents a novel occurrence. Other respiratory disorders that may occur in the perioperative period include narcotic intoxication, atelectasis, pneumonia, sepsis, PTE, cardiogenic heart failure, transfusion-associated circulatory overload (TACO), anaphylaxis, and asthma attacks. This case, in particular, required differentiation from TACO, anaphylaxis, and asthma. We ruled out anaphylaxis and asthma due to the absence of skin allergy symptoms, and the patient’s breathing pattern was not asthmatic. Cardiogenic pulmonary edema was excluded, and TRALI was suspected because left ventricular motion on echocardiogram was maintained before and after CPR, despite the rapid progression of pulmonary edema on chest X-ray (**Fig. 2**). PTE was ruled out because echocardiography revealed no right ventricular stress findings. In addition, the decreased NT-proBNP levels before and after transfusion indicated that the patient did not have cardiogenic pulmonary edema such as TACO. Myocardial infarction was also ruled out as the left ventricular motion on echocardiography was normal, and there were no findings of asynergy. Moreover, the flow of the gastroepiploic artery, which was the inflow of CABG, was patent. Furthermore, no postoperative increase was observed in the cardiac enzyme levels. The absence of other risk factors for ALI led us to the exclusive diagnosis of TRALI type I.

Various hypotheses exist for the mechanism of TRALI. Anti-leukocyte and anti-granulocyte antibodies have been the focus of attention.^3,4)^ The risk of TRALI may be reduced using male-predominant plasma transfusion, which has been introduced in major countries.^10)^

One of the major strategies to prevent the development of TRALI is to avoid blood transfusion if possible. This includes appropriate hemostatic techniques, optimal heparin doses, and protamine reversal when systemic heparinization is no longer required. Some alternative strategies are the use of a cell saver or collection of autologous blood transfusion before surgery. In this case, the most common causes of bleeding were preoperative thrombocytopenia and severe arterial calcification. Because the aneurysm was relatively large, we planned an early surgery and did not opt for autologous blood transfusion.

The standard treatment for TRALI is supportive care with oxygen and catecholamines. All patients require oxygen, and 70%–90% of patients require ventilator management.^3)^ This was a serious case in which the patient experienced respiratory failure and cardiac arrest immediately after the TRALI onset. However, TRALI was suspected at an early stage, and continuous adrenaline administration and ventilator management were initiated, which might have contributed to the patient’s survival.

## Conclusion

We successfully diagnosed and treated a case of TRALI type I that occurred immediately after AAA repair. Perioperative TRALI is difficult to identify in invasive surgery. Surgical cases requiring blood transfusion should be carefully monitored, and prompt action should be taken after the onset of symptoms indicating the possibility of TRALI.
